# BTNL2 Inhibits Pyroptosis in H37Ra-Infected Macrophages by Maintaining Mitochondrial Homeostasis

**DOI:** 10.3390/microorganisms14061188

**Published:** 2026-05-25

**Authors:** Yazhi Feng, Yiyao Liu, Guangxin Chen, Changxin Wu

**Affiliations:** 1 Key Laboratory of Medical Molecular and Cellular Biology of Shanxi Province, Institutes of Biomedical Sciences, Shanxi University, Taiyuan 030006, China; fengyazhi@sxu.edu.cn (Y.F.); liuyiyao@sxu.edu.cn (Y.L.); 2 Biomedicine and Health Laboratory in Shanxi Province, Shanxi University, Taiyuan 030006, China; 3 Key Laboratory of Chemical Biology and Molecular Engineering of Education Ministry, Shanxi University, Taiyuan 030006, China

**Keywords:** BTNL2, *Mycobacterium tuberculosis* H37Ra, pyroptosis, mitochondrial damage

## Abstract

Butyrophilin-like 2 (BTNL2) is an immunomodulatory molecule critically involved in regulating the host immune response to infection with the avirulent *Mycobacterium tuberculosis* strain H37Ra. However, its functional role in modulating pyroptosis and associated inflammatory responses remains incompletely characterized. Here, we demonstrate that BTNL2 deficiency exacerbates pyroptosis and the inflammatory response in H37Ra-infected murine peritoneal macrophages via two distinct pathways. First, the loss of BTNL2 induces excessive mitochondrial damage, which leads to aberrant release of mitochondrial DNA (mtDNA) and accumulation of mitochondrial reactive oxygen species (mtROS), thereby triggering NLRP3 (NOD-like receptor family pyrin domain containing 3) inflammasome activation and gasdermin D (GSDMD)-mediated pyroptosis. Second, cytosolic mtDNA accumulation hyperactivates the cGAS–STING signaling axis, resulting in transcriptional upregulation of NLRP3 and consequent amplification of pro-inflammatory cytokine production. Collectively, these findings demonstrate that BTNL2 acts as a regulator of mitochondrial homeostasis and innate immune balance during H37Ra infection in primary peritoneal macrophages. The results provide mechanistic insights into BTNL2 function in the context of H37Ra-induced pyroptosis.

## 1. Introduction

H37Ra is an avirulent laboratory strain widely used as a surrogate to study host–pathogen interactions [1,2]. As an obligate intracellular bacterium, H37Ra can survive within host macrophages for a prolonged period [3], making it a useful model for investigating the cellular mechanisms that govern intracellular clearance. Innate immunity serves as the host’s first line of defense against microbial pathogens, including H37Ra, by mounting rapid, non-specific immune responses that not only restrict initial infection but also shape subsequent adaptive immunity [4]. Effective control of H37Ra infection depends on the innate immune system’s capacity to recognize the bacterium and initiate appropriate antimicrobial effector functions. Pyroptosis, one type of programmed cell death of macrophages, plays a dual role in both limiting bacterium proliferation [5,6] and driving inflammatory responses [7]. Pyroptosis is a lytic form of cell death mediated by gasdermin family proteins, which relies on inflammasome assembly and subsequent activation of caspase-1 or caspase-11 [8]. H37Ra infection has been shown to specifically engage the NLRP3 (NOD-like receptor family pyrin domain containing 3) inflammasome pathway, resulting in gasdermin D (GSDMD) cleavage, pyroptotic cell death, and robust interleukin-1β (IL-1β) and interleukin-18 (IL-18) secretion [9].

Inflammation constitutes a fundamental component of the innate immune response. While acute and tightly regulated inflammatory responses are essential for effective pathogen clearance [10], their persistence or dysregulation can precipitate collateral tissue damage and thereby accelerate disease progression [11]. Consequently, precise spatiotemporal regulation of inflammation is indispensable for maintaining the delicate balance between protective immunity and immunopathology.

Mitochondria serve as central signaling hubs that integrate cellular energy metabolism with innate immune responses. Disruption of mitochondrial homeostasis, including cytosolic release of mitochondrial DNA (mtDNA) and excessive accumulation of mitochondrial reactive oxygen species (mtROS), directly activates the NLRP3 inflammasome [12], thereby amplifying NLRP3-dependent pyroptosis and pro-inflammatory cytokine production [9]. Concurrently, the cGAS–STING pathway serves as a critical cytosolic DNA-sensing mechanism that detects aberrant nucleic acid localization, whether of genomic or organelle-derived origin [13]. Upon mitochondrial damage-induced loss of membrane integrity, liberated mtDNA acts as a potent endogenous damage-associated molecular pattern (DAMP), triggering cGAS–STING activation and its downstream type I interferon and NF-κB signaling—responses that synergize with NLRP3 inflammasome activity to intensify pyroptotic cell death and inflammatory pathology [14,15]. Although accumulating evidence has clarified the individual contributions of mitochondrial dysfunction and cGAS–STING activation to pyroptosis regulation, the mechanisms by which cells preserve mitochondrial integrity to balance the defensive function of pyroptosis against inflammatory damage remain incompletely understood.

Butyrophilin-like 2 (BTNL2), a member of the butyrophilin (BTN) family exhibiting structural and functional homology to the B7 co-stimulatory molecule family, is an immunomodulatory molecule with well-documented roles in immune regulation [16]. Genetic and clinical evidence links BTNL2 dysregulation to several chronic inflammatory and autoimmune conditions, including sarcoidosis, idiopathic inflammatory myopathies, and TB [17,18,19]. There is increasing evidence that BTNL2 plays a key role in regulating inflammatory responses [20], and BTN-like molecules are increasingly recognized as tissue-specific modulators of innate immunity and inflammation [21]. However, whether BTNL2 contributes to inflammation control by maintaining mitochondrial homeostasis and inhibiting pathological pyroptosis remains unexplored.

To investigate the role of BTNL2 in pyroptosis during mycobacterial infection, we used the avirulent *Mycobacterium tuberculosis* strain H37Ra. We demonstrate that BTNL2 acts as a negative regulator of NLRP3 inflammasome activation by maintaining mitochondrial homeostasis and thereby preventing pathological mtDNA release and mtROS accumulation. Moreover, BTNL2 suppresses aberrant cGAS–STING pathway activation, thereby attenuating downstream type I interferon and NF-κB-driven inflammatory responses. Consequently, BTNL2 deficiency results in coordinated hyperactivation of both the NLRP3–pyroptosis and cGAS–STING signaling axes, leading to exacerbated inflammation and immunopathological damage during H37Ra infection. Collectively, these findings provide mechanistic insights into BTNL2 function in the context of H37Ra infection and suggest that modulating BTNL2 activity may influence mycobacterial infection-associated inflammatory responses, although the relevance to virulent *Mycobacterium tuberculosis* strains or tuberculosis pathogenesis requires further investigation.

## 2. Materials and Methods

### 2.1. Mtb H37Ra Culture

*Mtb* (H37Ra) was obtained from Gene Optimal Biotechnology Co., Ltd. (Shanghai, China) and cultured in Middlebrook 7H9 liquid medium (Becton Dickinson Biosciences, Franklin Lakes, NJ, USA) supplemented with 10% (*v*/*v*) OADC (Middlebrook, NY, USA), under aerobic conditions at 37 °C.

### 2.2. Isolation of Primary Peritoneal Macrophages from Mice

A volume of 3 mL of 3% (*w*/*v*) thioglycolate broth was injected intraperitoneally into wild-type (WT) and BTNL2 knockout (BTNL2^−/−^) mice. After 3 days, mice were euthanized, and 5 mL of ice-cold 0.9% sodium chloride solution was injected into the abdomen of mice, followed by a gentle abdominal massage for 5 min. The resulting lavage fluid containing primary macrophages was collected and centrifuged at 1000× *g* for 5 min at 4 °C. The cell pellet was resuspended in DMEM medium (Gibco, Waltham, MA, USA) supplemented with 5% (*v*/*v*) FBS (Sorfa, Huzhou, China) and 1% (*v*/*v*) penicillin–streptomycin solution (NCM Biotech, Suzhou, China). Cells were seeded into culture dishes and incubated for 6 h at 37 °C under 5% CO_2_. Non-adherent cells were then removed by gentle washing with PBS, and the remaining adherent cells were considered primary macrophages.

### 2.3. Quantitative Real-Time PCR (qRT-PCR)

Total RNA was isolated from cells using the Improved Tissue/Cell RNA Extraction Kit (Sparkjade, Shandong, China). First, cDNA synthesis was performed with the 5× All-In-One RT MasterMix (Abm, Richmond, BC, Canada), according to the manufacturer’s instructions. qRT-PCR was conducted using BlasTaq 2× qPCR MasterMix (Abm, Richmond, BC, Canada). Each 10 μL reaction mixture contained 5 μL of 2× qPCR MasterMix, 0.5 μL each of gene-specific forward and reverse primers, 2 μL of diluted cDNA template, and 2 μL of nuclease-free water. Thermal cycling conditions consisted of an initial denaturation at 95 °C for 5 min, followed by 35 cycles of denaturation at 95 °C for 15 s, annealing at 60 °C for 60 s, and extension at 72 °C for 30 s. The primer sequences used were as follows: NLRP3, F: 5′-GAGCTGGACCTCAGTGACAATGC-3′, R: 5′-ACCAATGCGAGATCCTGACAACAC-3′; IL-1β, F: 5′-TGCCACCTTTTGACAGTGATG-3′, R: 5′-TGATGTGCTGCTGCGAGATT-3′; IFN-β, F: 5′-GCCTTTGCCATCCAAGAGATGC-3′, R: 5′-ACACTGTCTGCTGGTGGAGTTC-3′; β-actin, F: 5′-GTCAGGTCATCACTATCGGCAAT-3′, R: 5′-AGAGGTCTTTACGGATGTCAACGT-3′. Relative gene expression was calculated using the 2^−ΔΔCT^ method with β-actin as the internal reference.

### 2.4. Flow Cytometry

Following infection with H37Ra at a multiplicity of infection (MOI) of 10 for 24 h, cells were washed twice with ice-cold PBS. Propidium Iodide (PI) (Solarbio, Beijing, China) was diluted 1:200 in serum-free DMEM and added to the monolayer for 30 min at 37 °C. Then, the cells were trypsinized and gently harvested, and PI-positive cells were analyzed by flow cytometry using an excitation wavelength of 488 nm.

### 2.5. Western Blot

Cells were lysed using RIPA Lysis Buffer (Beyotime, Shanghai, China), and total protein concentration was quantified using the Pierce BCA Protein Assay Kit (Thermo, Rockford, IL, USA). Protein samples were separated by SDS-PAGE gels at 110 V for 1.5 h, followed by electrotransfer onto PVDF membranes (Merck Millipore, Burlington, MA, USA) at 100 V for 1 h. Membranes were blocked with 5% non-fat milk in TBS-T for 2 h at room temperature. After washing with TBS-T, membranes were incubated with primary antibodies overnight at 4 °C. Following another TBS-T wash, membranes were incubated with HRP-conjugated secondary antibodies for 1 h at room temperature. Protein signals were detected using NcmECL Ultra Luminol (NCM Biotech, Suzhou, China). Primary antibodies included iNOS (CST, Danvers, MA, USA), GAPDH, HRP-conjugated secondary antibody (ABclonal, Wuhan, China), NLRP3 (Abcam, Cambridge, UK), ASC, Caspase-1, GSDMD (Bioss, Beijing, China), GSDMD-N, HO-1, P-STING (Immunoway, San Jose, CA, USA), COX-2, TXNIP (Affinity, Changzhou, China), β-actin (Abways, Shanghai, China), NRF2 (Bioswamp, Wuhan, China), BTNL2, cGAS (Proteintech, Wuhan, China), PINK1, Parkin, Atp5a1, VDAC1, STING, IRF3, and P-IRF3 (ZEN-BIO, Chengdu, China).

### 2.6. Hoechst 33342/PI Staining

Macrophages were stained with a Hoechst 33342/PI Double Staining Kit (Solarbio, Beijing, China). Following seeding the cells in a 24-well plate and infection with H37Ra for 0, 24, 48 and 72 h at an MOI of 10. Then, Hoechst 33342 and PI were diluted 1:200 in serum-free DMEM and used to incubate the cells for 30 min at 37 °C. Next, the cells were subsequently washed 3 times with ice-cold PBS and imaged using a fluorescence microscope (Nikon, Tokyo, Japan).

### 2.7. Cellular Immunofluorescence

Macrophages were seeded onto sterile glass coverslips placed in 24-well plates. Following infection with H37Ra at an MOI of 10 for 24 h, cells were washed 3 times with ice-cold PBS, 5 min per wash. Cells were then fixed with 4% (*w*/*v*) paraformaldehyde for 10 min at room temperature, followed by 3 additional PBS washes. Permeabilization was performed with 0.1% (*v*/*v*) Triton X-100 in PBS for 10 min at room temperature. Next, the cells were blocked with 5% (*w*/*v*) BSA in PBS for 1 h at room temperature. After blocking, the cells were incubated overnight at 4 °C with the primary antibody diluted 1:200 in PBS containing 5% BSA, and then washed with ice-cold PBS. The cells were then incubated for 1 h at room temperature with goat anti-rabbit IgG (H + L) highly cross-adsorbed (Bioswamp, Wuhan, China) and conjugated to Alexa Fluor™488 (Abcam, Cambridge, UK) diluted 1:2000 in PBS containing 5% BSA. Following another wash, the cells were stained with DAPI for 5 min. Finally, the coverslips were washed with ice-cold PBS and imaged using a fluorescence microscope.

### 2.8. ROS Quantification

Intracellular ROS levels in macrophages were assessed using a Reactive Oxygen Species Assay Kit (Solarbio, Beijing, China). DCFH-DA was diluted 1:1000 in serum-free DMEM. Following infection with H37Ra for 24 h at an MOI of 10, the culture medium was carefully replaced with the DCFH-DA solution and incubated at 37 °C for 30 min. Subsequently, cells were washed 3 times with ice-cold PBS. Finally, fluorescence microscopy was used to observe and capture images of the cells.

### 2.9. MDA Detection

Malondialdehyde (MDA) levels in macrophages were quantified using a commercial Malondialdehyde Assay Kit (Solarbio, Beijing, China). Following infection with H37Ra for 24 h at an MOI of 10, cells were harvested using an extraction solution. Cell lysis was performed using an ultrasonic processor set at 200 W output power, 3 s pulse duration, 10 s interval, and a total of 30 cycles to ensure complete membrane disruption. The lysate was then centrifuged at 8000× *g* for 10 min at 4 °C, and the clear supernatant was collected and stored on ice. According to the manufacturer’s protocol, the supernatant was combined with the appropriate reagents in a 1.5 mL EP tube. The mixture was incubated at 100 °C for 1 h. Subsequently, 200 μL of the sample was transferred to a 96-well plate, and absorbance readings were taken at 532 nm and 600 nm.

### 2.10. GSH Detection

The intracellular reduced glutathione (GSH) concentration in macrophages was quantified using a commercial Reduced Glutathione Assay Kit (Solarbio, Beijing, China) following the manufacturer’s instructions. Briefly, after macrophages were infected with H37Ra at an MOI of 10 for 24 h, cells were harvested and lysed in ice-cold GSH extraction buffer using probe sonication (200 W output power, 3 s pulse duration, 10 s interval, and a total of 30 cycles) to ensure complete membrane disruption. The lysates were centrifuged at 12,000× *g* for 10 min at 4 °C, and the supernatant was collected and assayed immediately to avoid GSH degradation. The supernatant was mixed with assay reagents in 1.5 mL EP tubes as specified by the kit protocol. Following a 2 min incubation at room temperature, 200 µL of each reaction mixture was transferred to a 96-well plate, and absorbance was measured at 412 nm using a microplate spectrophotometer.

### 2.11. LDH Activity Assay

Lactate dehydrogenase (LDH) activity in macrophages was quantified using the LDH Activity Assay Kit (Solarbio, Beijing, China), based on the manufacturer’s protocol. Briefly, after macrophages were infected with H37Ra at an MOI of 10 for 0, 24, 48 and 72 h, cells were harvested and lysed in ice-cold LDH extraction buffer via probe sonication (200 W output power; 3 s pulse duration, 10 s interval, 30 cycles) to ensure complete membrane disruption. The lysates were centrifuged at 8000× *g* for 10 min at 4 °C, and the clear supernatant was collected. Each supernatant was mixed with assay reagents in a 1.5 mL EP tube at the ratio specified by the kit. After incubation at 37 °C, 200 µL of the reaction mixture was transferred to a 96-well plate, and absorbance was measured at 450 nm using a microplate spectrophotometer.

### 2.12. Detection of mtROS

Mitochondrial mtROS in macrophages were detected using MitoSOX Red mitochondrial superoxide indicator (Thermo Fisher Scientific, Waltham, MA, USA). MitoSOX Red was diluted to a final concentration of 500 Nm in serum-free DMEM medium. After infection with H37Ra at an MOI of 10 for 24 h, the culture medium was replaced with the MitoSOX Red solution and incubated at 37 °C for 30 min. Subsequently, cells were washed 3 times with ice-cold PBS and observed and imaged using fluorescence microscopy.

### 2.13. ELISA Assay

Mouse IL-1β and IL-18 concentrations in macrophage culture supernatants were quantified using commercially available ELISA kits (ELK, Wuhan, China), according to the manufacturer’s instructions. Briefly, after macrophages were infected with H37Ra at an MOI of 10 for 0, 24, 48 and 72 h, supernatant was collected and centrifuged at 4000× *g* for 10 min at 4 °C to remove cellular debris. The supernatants were loaded onto a 96-well ELISA plate. Subsequently, 100 µL of HRP-conjugated detection antibody was added to each well, and plates were incubated at 37 °C for 60 min in a humidified chamber. After incubation, plates were washed 5 times with wash buffer, each for 1 min. Next, 50 µL of chromogen solution A and 50 µL of chromogen solution B were added to each well, gently mixed and incubated at 37 °C for 15 min. Finally, 50 µL of stop solution was added to each well, and absorbance was measured at 450 nm using a microplate spectrophotometer.

### 2.14. Isolation of Cellular Mitochondria

Mitochondria were isolated from macrophages using the Cell Mitochondria Isolation Kit (Beyotime Biotechnology, Beijing, China), according to the manufacturer’s instructions. Briefly, macrophages were infected with H37Ra at an MOI of 10 for 24 h. Subsequently, cells were harvested by centrifugation at 300× *g* for 5 min at 4 °C, washed twice with ice-cold PBS, and resuspended in ice-cold mitochondrial isolation reagent (1 mL per 2 × 10^7^ cells). The cell suspension was homogenized using a glass homogenizer for 30 strokes. The homogenate was centrifuged at 600× *g* for 10 min at 4 °C to remove unbroken cells and nuclei, and the resulting supernatant was carefully collected and centrifuged at 11,000× *g* for 10 min at 4 °C. The resulting pellet represented the mitochondrial fraction, and the supernatant was retained as the cytosolic fraction.

### 2.15. Quantification of mtDNA

mtDNA was extracted from the purified mitochondrial fraction using the Universal Genomic DNA Extraction Kit (CWBiotech, Taizhou, Jiangsu, China), in accordance with the manufacturer’s instructions. qRT-PCR was performed to quantify the relative abundance of mitochondrial 16S ribosomal RNA (16S rRNA) and NADH:ubiquinone oxidoreductase core subunit 4 (ND4). Amplification reactions were carried out using BlasTaq 2× qPCR Master Mix (Abm, Richmond, BC, Canada). Each 10 µL reaction mixture consisted of 5 µL of 2× master mix, 0.5 µL of forward primer, 0.5 µL of reverse primer, 2 µL of template DNA, and 2 µL of nuclease-free water. Thermal cycling was conducted as follows: initial denaturation at 95 °C for 5 min; followed by 35 cycles of denaturation at 95 °C for 15 s, annealing at 60 °C for 60 s, and extension at 72 °C for 30 s. The primer sequences used were as follows: 16s—F: 5′-CACTGCCTGCCCAGTGA-3′, R: 5′-ATACCGCGGCCGTTAAA-3′; ND4—F: 5′-AACGGATCCACAGCCGTA-3′, R: 5′-AGTCCTCGGGCCATGATT-3′; Tert—F: 5′-CTAGCTCATGTGTCAAGACCCTCTT-3′, R: 5′-GCCAGCACGTTTCTCTCGTT-3′. Relative gene expression was calculated using the 2−^ΔΔCT^ method, with Tert as the internal reference gene.

### 2.16. MitoTacker Staining

Mitochondrial morphology in macrophages was assessed by fluorescence microscopy following staining with MitoTracker (CST, Danvers, MA, USA). The probe was diluted to a final concentration of 300 nM in serum-free DMEM medium. After infection with H37Ra at an MOI of 10 for 24 h, the culture medium was replaced with the diluted MitoTacker solution and incubated for 30 min at 37 °C in a humidified 5% CO_2_ incubator. Subsequently, cells were washed 2 times with ice-cold PBS to remove unbound probe. Fluorescence images were acquired using a fluorescence microscope.

### 2.17. Detection of Proteins in Cell Culture Supernatant

Cell culture supernatants were concentrated using centrifugal filter devices with regenerated cellulose membranes and a nominal molecular weight cutoff of 3 kDa (Merck Millipore, Darmstadt, Germany), following the manufacturer’s instructions. Briefly, macrophages were infected with H37Ra at an MOI of 10 for 24 h. After infection, supernatants were collected and centrifuged at 4000× *g* for 10 min at 4 °C to remove cellular debris. The clarified supernatant was then loaded onto centrifugal filtration units and concentrated by centrifugation at 12,000× *g* for 10 min at 4 °C. Subsequently, the inner collection tube containing the retentate was carefully inverted into a fresh, low-protein-binding outer tube and subjected to a brief recovery spin at 1000× *g* for 5 min at 4 °C. The eluate recovered in the outer tube constituted the concentrated supernatant protein fraction.

### 2.18. Statistical Analysis

The data in the manuscript was presented as mean ± SEM. *t*-test was used to analyze the significant differences (* *p* < 0.05, ** *p* < 0.01, *** *p* < 0.001, and **** *p* < 0.0001). All experiments were carried out with three independent replications.

## 3. Results

### 3.1. BTNL2 Inhibits Cell Death and NLRP3 Inflammasome-Related Gene Expression

**Figure 1:**
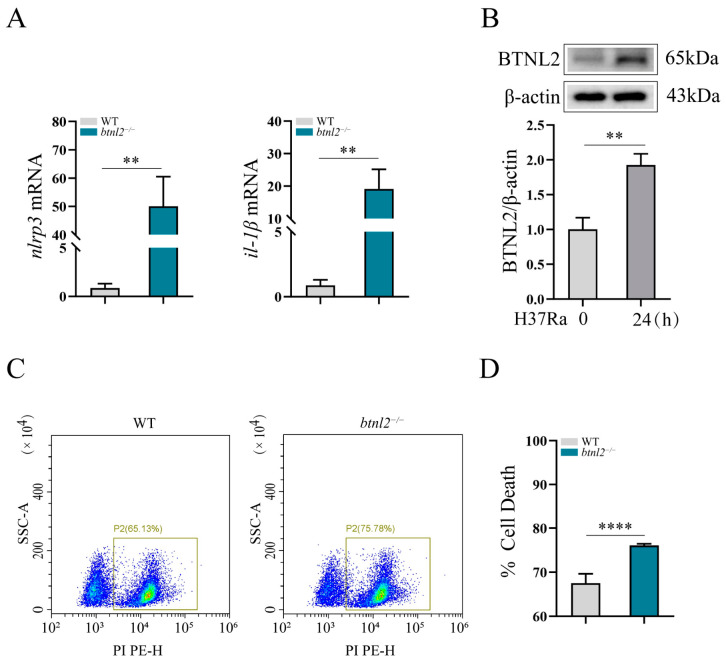
BTNL2 inhibits cell death and NLRP3 inflammasome-related gene expression. (**A**) qRT-PCR analyzed the expression of *nlrp3* and *il-1β* mRNA in primary peritoneal macrophages from WT and BTNL2^−/−^ mice (*n* = 3); (**B**) WB analyzed the expression of BTNL2 in H37Ra-infected primary peritoneal macrophages from WT mice (*n* = 3); (**C**,**D**) Flow cytometric quantification of propidium iodide (PI)-positive cells in WT and BTNL2^−/−^ peritoneal macrophages (*n* = 3). Means ± SEM, ** *p* < 0.01 and **** *p* < 0.0001 represent a significant difference.

Compared to WT mice, qRT-PCR analysis revealed significant upregulation of *nlrp3* and *il-1β* mRNA levels in primary peritoneal macrophages isolated from BTNL2^−/−^ mice (Figure 1A). The expression of BTNL2 in mice peritoneal macrophages exhibited an upward trend post H37Ra infection (Figure 1B). Flow cytometry analyses showed that, post H37Ra infection, BTNL2^−/−^ macrophages exhibited a significantly higher rate of cell death compared with WT macrophages (Figure 1C,D), and this death was not caused by apoptosis (Figure S1). These findings indicate that BTNL2 deficiency not only exacerbates infection-associated cytotoxicity but also augments NLRP3 inflammasome activation and subsequent pro-inflammatory cytokine production.

### 3.2. BTNL2 Inhibits H37Ra Infection-Induced Pyroptosis in Macrophages

**Figure 2:**
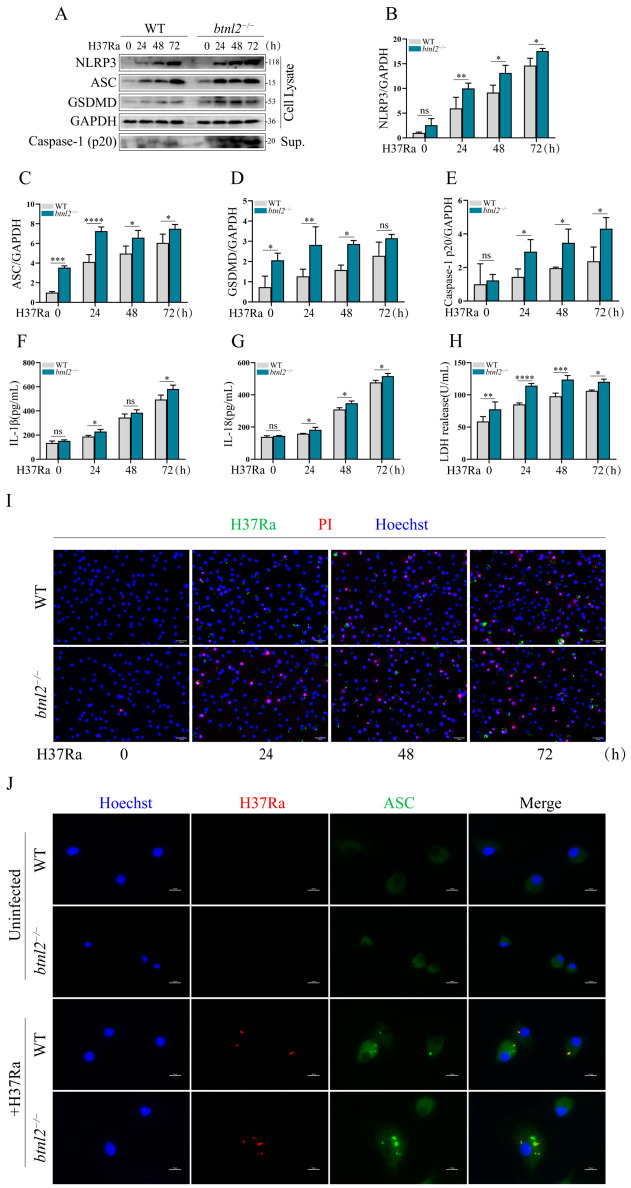
BTNL2 inhibits H37Ra infection-induced pyroptosis in macrophages. Peritoneal macrophages from WT and BTNL2^−/−^ mice were infected with H37Ra at an MOI of 10 for 0, 24, 48 and 72 h. (**A**–**E**) WB analyzed the expression of NLRP3, ASC, Caspase-1 (P20), and GSDMD in H37Ra-infected primary peritoneal macrophages from WT and BTNL2^−/−^ mice (*n* = 3); (**F**,**G**) ELISA analyzed the IL-1β and IL-18 in H37Ra-infected primary peritoneal macrophages from WT and BTNL2^−/−^ mice (*n* = 3); (**H**) The LDH activity assay kit was used to analyze the LDH activity in H37Ra-infected primary peritoneal macrophages from WT and BTNL2^−/−^ mice (*n* = 3); (**I**) Hoechst 33342/PI dual staining analyzed the pyroptosis in H37Ra-infected primary peritoneal macrophages from WT and BTNL2^−/−^ mice, green represents H37Ra, red represents PI staining, blue represents Hoechst 33342 staining, the images are 20×, and the scale bar is 100 μm (*n* = 3); (**J**) Immunofluorescence analyzed the staining for ASC specks in H37Ra-infected primary peritoneal macrophages from WT and BTNL2^−/−^ mice, the images are 150×, and the scale bar is 10 μm (*n* = 3); means ± SEM, * *p* < 0.05, ** *p* < 0.01, *** *p* < 0.001, and **** *p* < 0.0001 represent a significant difference. ns, not significant.

To investigate whether BTNL2 deficiency exacerbates pyroptosis in peritoneal macrophages, we employed H37Ra—a well-characterized physiological activator of NLRP3-dependent pyroptosis—to induce pyroptosis and assess the effects of BTNL2 during H37Ra infection. Peritoneal macrophages from WT and BTNL2^−/−^ mice were infected with H37Ra for 0, 24, 48 and 72 h. Western blot analysis revealed time-dependent upregulation of key NLRP3 inflammasome components, including NLRP3, ASC, Caspase-1 (p20) and GSDMD, in the macrophages from BTNL2^−/−^ mice in comparison with that in the macrophages from WT mice (Figure 2A–E). Consistent with enhanced inflammasome activation, ELISA assays demonstrated markedly increased secretion of mature IL-1β and IL-18 in BTNL2^−/−^ macrophages relative to WT controls (Figure 2F,G). Similarly, LDH activity measurement assays confirmed significantly greater plasma membrane permeabilization in BTNL2^−/−^ macrophages following H37Ra infection (Figure 2H). These findings were corroborated by Hoechst 33342/propidium iodide (PI) dual staining, which showed a significant increase in PI-positive cells in the BTNL2^−/−^ macrophage population at 24, 48 and 72 h post-infection (Figure 2I). Notably, the peak of pyroptosis occurred at 24 h post-infection, consistent with a previous study [22]. To further interrogate inflammasome assembly at this critical time point, immunofluorescence microscopy was performed to detect ASC speck formation. The results indicated that BTNL2^−/−^ macrophages displayed a significantly higher frequency of ASC speck-positive cells at 24 h post-infection (Figure 2J), providing direct evidence of augmented NLRP3 inflammasome oligomerization in the absence of BTNL2.

### 3.3. BTNL2 Inhibits H37Ra-Induced Oxidative Stress

**Figure 3:**
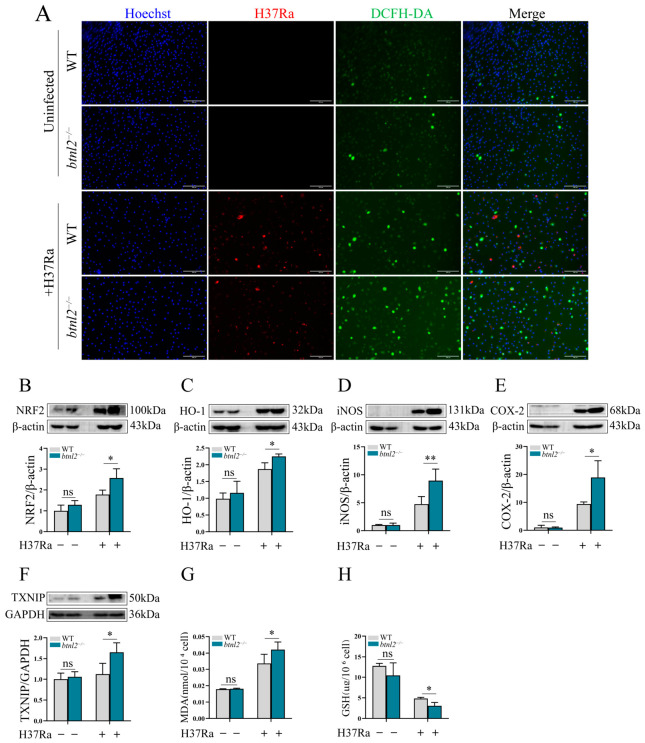
BTNL2 inhibits H37Ra-induced oxidative stress. Peritoneal macrophages from WT and BTNL2^−/−^ mice were infected with H37Ra at an MOI of 10 for 0 and 24 h, + represents H37Ra infection, and – represents no infection. (**A**) The Reactive Oxygen Species Assay Kit was used to measure the production of ROS in H37Ra-infected macrophages from WT and BTNL2^−/−^ mice; blue represents Hoechst 33342, green represents ROS, red represents H37Ra, the images are 20×, and scale bar is 100 μm (*n* = 3); (**B**–**F**) WB analyzed the expression of NRF2, HO-1, iNOS, COX-2 and TXNIP in H37Ra-infected macrophages from WT and BTNL2^−/−^ mice (*n* = 3); (**G**,**H**) The MDA Content Assay Kit and Reduced Glutathione (GSH) Content Assay Kit were used to analyze the content of MDA and GSH in H37Ra-infected macrophages from WT and BTNL2^−/−^ mice (*n* = 3). Means ± SEM, * *p* < 0.05 and ** *p* < 0.01 represent a significant difference. ns, not significant.

Intracellular oxidative stress denotes a pathological condition characterized by an imbalance between the intracellular accumulation of ROS and other free radicals and the capacity of endogenous antioxidant defense systems, which can result in cellular damage and functional impairment [23]. Accumulating evidence indicates that, while ROS exhibit antimicrobial activity [24], their excessive accumulation also functions as a critical redox signal that triggers NLRP3 inflammasome activation. Subsequently, this promotes caspase-1 activation and GSDMD cleavage, culminating in pyroptosis and dysregulated inflammatory responses [25,26]. Furthermore, excessive ROS also induces thioredoxin-interacting protein (TXNIP) to dissociate from thioredoxin and binds directly to NLRP3, thereby facilitating NLRP3 inflammasome assembly and activation [27]. To investigate the effect of BTNL2 on oxidative stress during H37Ra infection, we quantified multiple markers of oxidative stress in infected macrophages. At 24 h post-H37Ra infection, BTNL2^−/−^ macrophages exhibited significantly higher intracellular ROS levels compared with WT controls (Figure 3A). Meanwhile, protein expression levels of the antioxidant regulators NRF2 and heme oxygenase-1 (HO-1) were upregulated in BTNL2^−/−^ macrophages, accompanied by increased expression of the pro-inflammatory mediator COX-2 and iNOS (Figure 3B–E). Notably, TXNIP expression was significantly elevated in BTNL2^−/−^ peritoneal macrophages (Figure 3F). Furthermore, BTNL2 deficiency led to a significant increase in MDA levels—a well-known biomarker of lipid peroxidation—alongside a marked reduction in GSH content (Figure 3G,H). Collectively, these results indicated that BTNL2 functions as a negative regulator of oxidative stress triggered by H37Ra infection.

### 3.4. BTNL2 Inhibits H37Ra-Induced Mitochondrial Dysfunction

**Figure 4:**
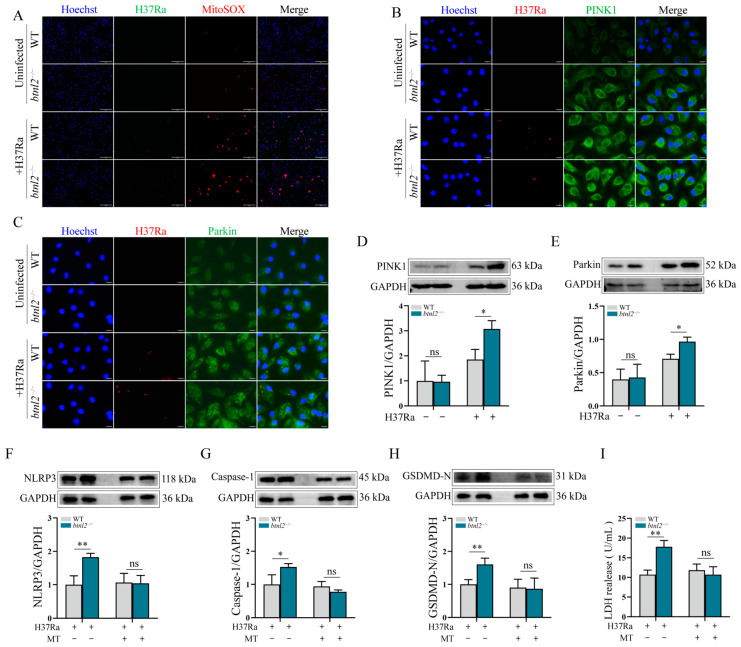
BTNL2 inhibits H37Ra-induced mitochondrial dysfunction. Peritoneal macrophages from WT and BTNL2^−/−^ mice were infected with H37Ra at an MOI of 10 for 0 and 24 h, + represents H37Ra infection, and – represents no infection. (**A**) The mitochondrial Reactive Oxygen Species Assay Kit was used to measure the generation of mtROS in H37Ra-infected macrophages from WT and BTNL2^−/−^ mice; blue represents Hoechst 33342, green represents H37Ra, red represents mtROS, the images are 20×, and scale bar is 100 μm (*n* = 3); (**B**,**C**) Immunofluorescence analyzed the expression of PINK1 and Parkin in H37Ra-infected macrophages from WT and BTNL2^−/−^ mice; blue represents Hoechst 33342, red represents H37Ra, green represents PINK1 or Parkin, the images are 150×, and scale bar is 10 μm (*n* = 3); (**D**,**E**) WB analyzed the expression of PINK1 and Parkin in H37Ra-infected macrophages from WT and BTNL2^−/−^ mice (*n* = 3); (**F**–**H**) The infected macrophages were treated with MitoTEMPO (MT) for 24 h, then WB was used to analyze the expression of NLRP3, Caspase-1, and GSDMD-N in H37Ra-infected macrophages from WT and BTNL2^−/−^ mice; (**I**) The infected macrophages were treated with MitoTEMPO (MT) for 24 h, then the LDH Activity Assay Kit was used to analyze the LDH activity in H37Ra-infected macrophages from WT and BTNL2^−/−^ mice, + represents MT treatment, and – represents no treatment. (*n* = 3). Means ± SEM, * *p* < 0.05 and ** *p* < 0.01 represent a significant difference. ns, not significant.

Mitochondria not only serve as the central organelles for cellular energy metabolism but also as the predominant intracellular source of ROS [28]. Excessive ROS generation can induce structural alterations in mitochondrial morphology and trigger mitochondrial dysfunction [29,30]. To determine whether BTNL2 deficiency exacerbates mtROS accumulation by disrupting mitochondrial homeostasis during H37Ra infection, we employed MitoSOX Red staining and found that BTNL2-deficient macrophages exhibited significantly elevated mtROS levels following H37Ra infection (Figure 4A). Furthermore, our results demonstrated that BTNL2-deficient mice showed a significant increase in the levels of PINK1 and Parkin in H37Ra-infected macrophages (Figure 4B–E). Prior studies have established that excessive mtROS contribute to oxidative stress amplification and promote NLRP3 inflammasome activation [31]. To test the hypothesis that BTNL2 suppresses pyroptosis primarily through preservation of mitochondrial function, we pharmacologically rescued mitochondrial dysfunction using the mitochondria-targeted antioxidant MitoTEMPO. Consistent with this hypothesis, MitoTEMPO treatment abrogated the protective effect of BTNL2 on mitochondrial homeostasis (Figure 4F–I), indicating that BTNL2-mediated inhibition of pyroptosis is mechanistically contingent upon its regulation of mitochondrial redox homeostasis.

### 3.5. BTNL2 Inhibits the Activation of cGAS-STING Signaling Pathway by Maintaining Mitochondrial Homeostasis

**Figure 5:**
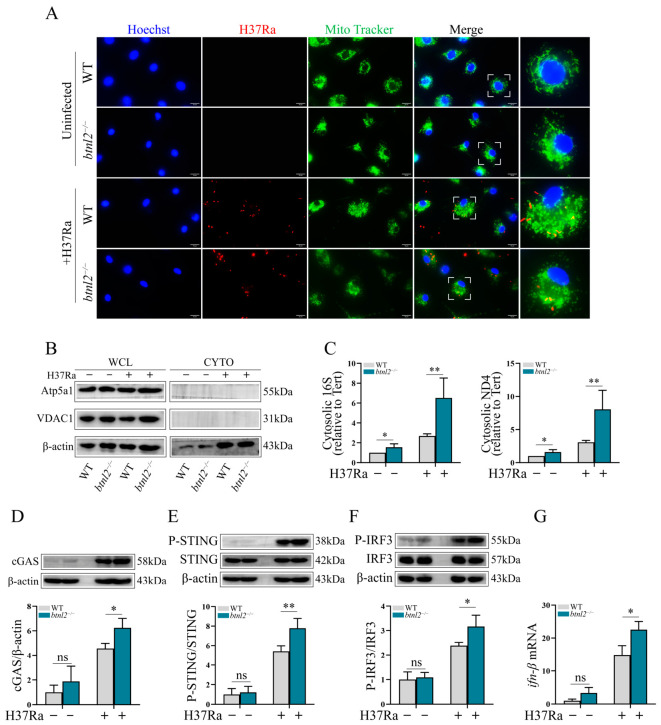
BTNL2 inhibits the activation of cGAS-STING signaling pathway by maintaining mitochondrial homeostasis. Peritoneal macrophages from WT and BTNL2^−/−^ mice were infected with H37Ra at an MOI of 10 for 0 and 24 h, + represents H37Ra infection, and – represents no infection. (**A**) Macrophages were stained with MitoTracker to assess mitochondrial morphology; blue represents Hoechst 33342, red represents H37Ra, green represents MitoTracker, white boxes represents the areas selected for higher magnification views shown in the adjacent panels, the images are 150×, and scale bar is 10 μm (*n* = 3); (**B**) Total and cytosolic fractions isolated from macrophages after infected with H37Ra for 0 and 24 h, and WB were used to analyze the mitochondrial proteins Atp5a1and VDAC1; (**C**) qPCR of mtDNA (16s and ND4) from cytosolic fractions in (**B**), quantified relative to total nuclear DNA (Tert) in H37Ra-infected macrophages; (**D**–**F**) WB analyzed the expression of cGAS, P-STING, STING, P-IRF3, and IRF3 in H37Ra-infected macrophages (*n* = 3); (**G**) qRT-PCR was used to analyze the expression of *ifn-β* mRNA in macrophages from WT and BTNL2^−/−^ mice (*n* = 3). Means ± SEM, * *p* < 0.05 and ** *p* < 0.01 represent a significant difference. ns, not significant.

Mitochondria commonly display a tubular morphology, and the dynamic equilibrium between fusion and fission is essential for preserving mitochondrial structural integrity [32]. Moreover, mitochondrial architecture and morphology serve as indicators of functional status [33,34]. To investigate whether BTNL2 deficiency disrupts mitochondrial morphology during H37Ra infection, we stained the H37Ra-infected macrophages from BTNL2^−/−^ mice with MitoTracker and observed significantly increased mitochondrial fragmentation compared with WT controls (Figure 5A), suggesting that BTNL2 is required for maintaining mitochondrial homeostasis under H37Ra infection conditions. mtDNA, which is normally confined to the mitochondrial matrix [35], can be released into the cytosol following mitochondrial damage, thereby activating innate immune sensors such as the NLRP3 inflammasome [36]. Consistent with the observed fragmentation, BTNL2 deficiency also promoted mtDNA release into the cytosol (Figure 5B–C). Furthermore, cytosolic mtDNA is sensed by the DNA receptor cGAS, leading to the induction of Interferon-β (IFN-β) and subsequent amplification of inflammatory responses [37]. Western blot analysis revealed that BTNL2 deficiency resulted in hyperactivation of the cGAS-STING signaling axis, accompanied by elevated IFN-β secretion (Figure 5D–G). Collectively, these findings demonstrate that BTNL2 deficiency directly drives mitochondrial fragmentation, thereby triggering cGAS–STING pathway activation and potentiating downstream inflammatory signaling. These findings indicated that BTNL2 functions to restrain cGAS–STING signaling by maintaining mitochondrial homeostasis.

## 4. Discussion

During infection, the innate immune system serves as the host’s primary defense against pathogen invasion and plays a pivotal role in containing early bacterial replication while orchestrating the development of adaptive immunity [38]. This coordinated response involves various immune cells, molecules, and complex regulatory mechanisms. As an obligate intracellular pathogen, H37Ra actively manipulates host cell death pathways, including pyroptosis, a lytic, pro-inflammatory form of programmed cell death characterized by gasdermin-mediated plasma membrane pore formation and subsequent release of IL-1β and IL-18 [9]. The role of pyroptosis in H37Ra infection is characterized by functional duality and mechanistic complexity. In macrophages and other myeloid cells, controlled pyroptosis serves as a critical antimicrobial defense: it disrupts the intracellular replicative niche of the bacteria and promotes recruitment and activation of additional immune effectors via cytokine release [39]. However, dysregulated or excessive pyroptosis can trigger pathological hyperinflammation, leading to widespread tissue damage and immunopathology [40], which may paradoxically impair bacterial clearance and promote bacterial persistence. Therefore, the host must maintain a balance between controlling bacterial infection and avoiding excessive inflammatory damage.

BTNL2 is a B7 family-related immunoregulatory molecule, and its primary function is the suppression of T cell activation and pro-inflammatory cytokine production [17,41]. Given this potent immunoinhibitory activity, BTNL2 is postulated to contribute critically to the fine-tuning of adaptive immune responses during *Mtb* infection. Supporting this hypothesis, genetic association studies have linked BTNL2 polymorphisms with altered susceptibility to TB. Although individual single-nucleotide polymorphisms (SNPs) in the BTNL2 locus failed to reach genome-wide significance in Chinese cohorts, specific haplotypes demonstrated robust statistical associations with disease risk [18]. While the precise molecular mechanisms remain to be fully elucidated, evidence supports a functional role for BTNL2 in the immunoregulation of *Mtb* infection.

In this study, we demonstrated that BTNL2 deficiency exacerbates pyroptosis in H37Ra-infected macrophages. Moreover, BTNL2 loss triggers robust activation of the canonical NLRP3 inflammasome pathway, evidenced by enhanced ASC speck formation and increased cleavage of caspase-1 and GSDMD. This cascade culminates in excessive secretion of IL-1β and IL-18, pronounced plasma membrane permeabilization, and significantly elevated macrophage cell death. In summary, our findings indicated that BTNL2 inhibits pyroptosis during H37Ra infection.

BTNL2 inhibits pyroptosis during H37Ra infection, yet the exact molecular mechanism behind this protective role remains unclear. Previous research has shown that bacterial infection triggers robust intracellular ROS accumulation, leading to oxidative stress [42]. While moderate ROS levels support antimicrobial defense and bacterial clearance, excessive ROS disrupts redox homeostasis, exacerbates inflammatory tissue injury, and paradoxically promotes bacterial persistence [43]. ROS serve as the primary mediators of oxidative stress; excessive ROS accumulation disrupts redox homeostasis and induces a state of oxidative stress, which constitutes a well-established upstream trigger of pyroptosis [44]. Since the documented role of ROS as critical initiators of pyroptotic signaling, we hypothesize that BTNL2 may attenuate pyroptosis through the suppression of oxidative stress.

Our findings demonstrated that, during H37Ra infection, BTNL2 deficiency increases intracellular oxidative stress, as evidenced by increased ROS accumulation; elevated expression of the antioxidant regulators NRF2 and HO-1, as well as the pro-oxidant enzymes iNOS and COX-2; higher levels of the lipid peroxidation marker MDA; and reduced GSH content. Collectively, these data indicated that BTNL2 functions to restrain oxidative stress in H37Ra-infected macrophages. However, whether BTNL2 attenuates H37Ra-induced pyroptosis through suppression of oxidative stress remains to be determined. Notably, BTNL2 deficiency markedly increased the expression of TXNIP, which serves as a key redox-sensitive adaptor that bridges oxidative stress and pyroptosis [45]. Under elevated ROS conditions, TXNIP dissociates from thioredoxin and directly binds NLRP3, thereby nucleating inflammasome assembly, triggering caspase-1-dependent pyroptosis, and amplifying downstream inflammatory responses [46]. Given that BTNL2 deficiency significantly enhanced TXNIP expression, our results suggest a mechanistic link whereby BTNL2 may mitigate pyroptosis in H37Ra-infected macrophages via modulation of the ROS–TXNIP–NLRP3 axis.

Mitochondria serve not only as the principal site of cellular energy production but also as the major intracellular source of ROS [47]. During H37Ra infection, BTNL2 deficiency significantly enhanced mtROS generation and elevated mtROS, which, in turn, amplifies global intracellular ROS accumulation, thereby triggering oxidative stress and initiating pyroptosis via the ROS–TXNIP–NLRP3 signaling axis. However, pharmacological inhibition of mtROS markedly attenuated pyroptosis in BTNL2^−/−^ macrophages, supporting the model that BTNL2 suppresses pyroptosis, at least in part by restraining mtROS production. Moreover, BTNL2 deficiency upregulated the expression of PINK1 and parkin during H37Ra infection, suggesting a functional role for BTNL2 in maintaining mitochondrial homeostasis. Consistently, BTNL2-deficient macrophages exhibited increased release of mtDNA fragments into the cytosol and robust activation of the cGAS–STING–IRF3 signaling pathway; notably, IRF3 activation directly induces NLRP3 transcription [48], which aids NLRP3 inflammasome assembly and the onset of pyroptosis.

**Figure 6:**
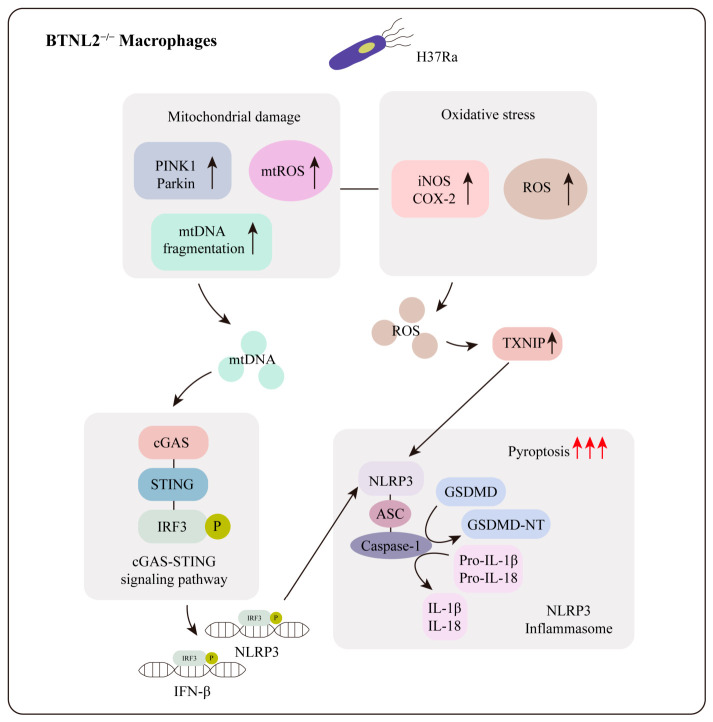
BTNL2 deficiency increases the onset of pyroptosis during H37Ra infection.

In summary, BTNL2 plays a critical negative regulatory role in modulating pyroptosis during H37Ra infection. During infection, BTNL2 deficiency triggers mitochondrial damage and dysfunction, leading to the accumulation of mtROS in mitochondria and the secretion of mtDNA fragmentation. mtROS accumulation increases the generation of more ROS and induces the onset of oxidative stress. ROS directly promotes pyroptosis via the TXNIP-NLRP3 axis. Furthermore, the release of mtDNA fragments induces the activation of cGAS/STING/IRF3 pathway and the expression of NLRP3 inflammasome. These findings reveal the essential role of BTNL2 as an immune checkpoint molecule in maintaining mitochondrial homeostasis and suppressing excessive inflammation mediated by H37Ra-induced pyroptosis (Figure 6).

## 5. Conclusions

In conclusion, our study identifies BTNL2 as a negative regulator of pyroptosis during H37Ra infection in primary peritoneal macrophages. BTNL2 deficiency exacerbates H37Ra-induced pyroptosis by promoting mitochondrial damage and dysfunction, leading to excessive accumulation of ROS and consequent oxidative stress. Mechanistically, elevated ROS drives pyroptosis through two synergistic pathways: (i) activation of the TXNIP–NLRP3 inflammasome axis, and (ii) induction of mitochondrial DNA (mtDNA) release into the cytosol, which hyperactivates the cGAS–STING signaling pathway and amplifies downstream inflammatory responses. Collectively, these findings establish BTNL2 as an immune negative regulatory factor that preserves mitochondrial homeostasis and constrains pyroptosis-associated inflammation during H37Ra infection in this in vitro model.

## Data Availability

All data are included in the manuscript.

## Supplementary Materials

The following supporting information can be downloaded at: https://www.mdpi.com/article/10.3390/microorganisms14061188/s1, Figure S1: BTNL2 deficiency does not affect H37Ra-induced apoptosis.

## Abbreviations

The following abbreviations are used in this manuscript:

BTN	Butyrophilin
BTNL2	Butyrophilin-like 2
DAMP	Damage-associated molecular pattern
GSDMD	Gasdermin D
GSH	Glutathione
HO-1	Heme oxygenase-1
IL-1β	Interleukin-1 beta
IL-18	Interleukin-18
IFN-β	Interferon-beta
LDH	Lactate dehydrogenase
MDA	Malondialdehyde
MOI	Multiplicity of infection
MT	MitoTEMPO
*Mtb*	*Mycobacterium tuberculosis*
mtDNA	Mitochondrial DNA
mtROS	Mitochondrial Reactive Oxygen Species
ND4	NADH Dehydrogenase Subunit 4
NLRP3	NOD-like receptor family pyrin domain containing 3
PI	Propidium Iodide
ROS	Reactive oxygen species
SNP	Single-nucleotide polymorphism
TB	Tuberculosis
TXNIP	Thioredoxin-interacting protein
16S rRNA	Mitochondrial 16S ribosomal RNA
