# Supplementary material for: BTNL2 Inhibits Pyroptosis in H37Ra-Infected Macrophages by Maintaining Mitochondrial Homeostasis
Source: Microorganisms. 2026 May 25;14(6):1188. doi: 10.3390/microorganisms14061188 (PMC13304222; doi:10.3390/microorganisms14061188)
Supplement: Supplementary file 1 [file microorganisms-14-01188-s001.zip › microorganisms-4288875-supplementary file.pdf]

Supplementary figures

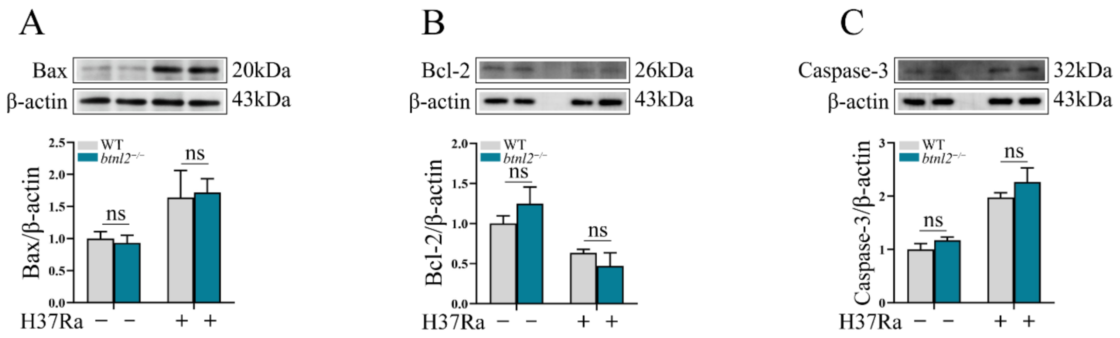

Figure S1. BTNL2 deficiency does not affect H37Ra-induced apoptosis. (A-C) WB analyzed the expression of Bax, Bcl-2 and Caspase-3 in H37Ra-infected primary peritoneal macrophages from WT and BTNL2 $^{-/-}$ mice (n = 3). ns, not significant.
